# Supplementary material for: Endothelial dysfunction and low-grade inflammation in the transition to renal replacement therapy
Source: PLoS One. 2019 Sep 13;14(9):e0222547. doi: 10.1371/journal.pone.0222547 (PMC6743867; doi:10.1371/journal.pone.0222547)
Supplement: S1 Fig — (DOCX) [file pone.0222547.s001.docx]

S1 Fig. Flowchart depicting the derivation of the cross-sectional and longitudinal study populations


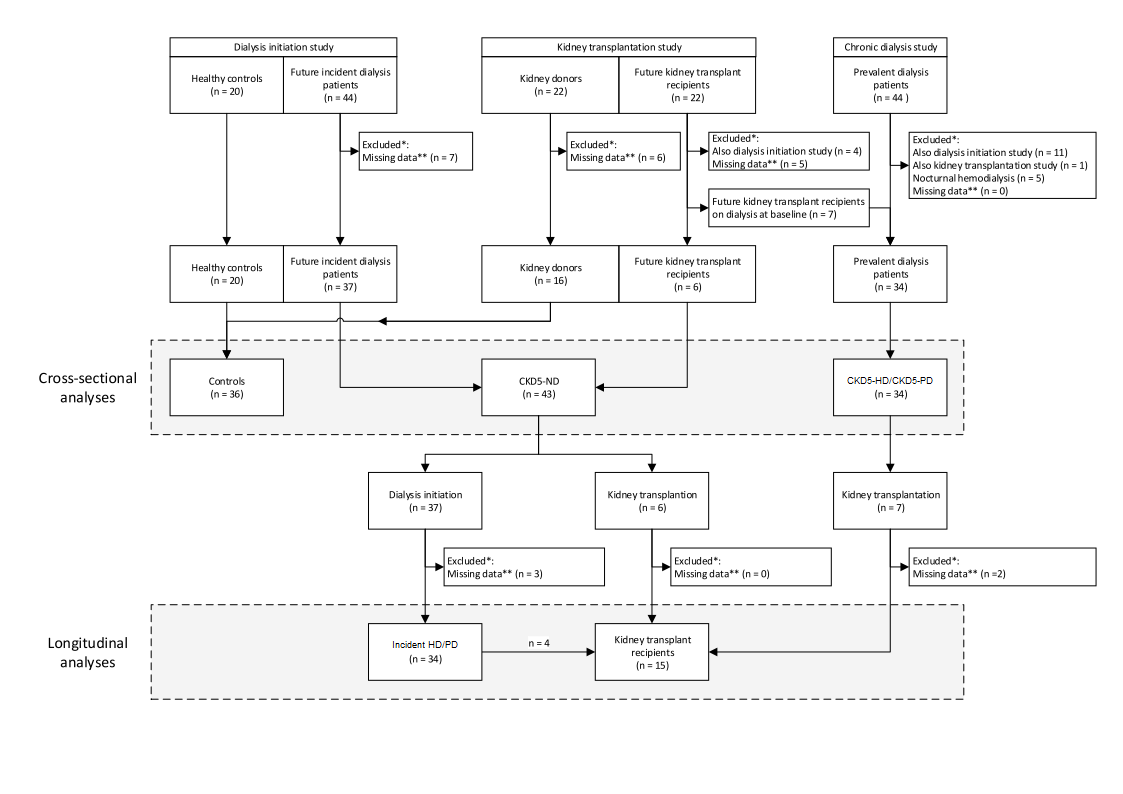


S1 Fig. Flowchart depicting the derivation of the cross-sectional and longitudinal study populations. Abbreviations: CKD5-HD, chronic kidney disease stage 5 hemodialysis; CKD5-ND, chronic kidney disease stage 5 non-dialysis; CKD5-PD, chronic kidney disease stage 5 peritoneal dialysis; HD, hemodialysis; PD, peritoneal dialysis. * Participants were sequentially excluded for the reasons described in the figure (*i.e.* counts are mutually exclusive), ** Missing data indicates absence of data on serum biomarkers of endothelial dysfunction and low-grade inflammation at baseline for the cross-sectional analyses or on at least one time point for the longitudinal analyses.
