# Supplementary material for: Endothelial dysfunction and low-grade inflammation in the transition to renal replacement therapy
Source: PLoS One. 2019 Sep 13;14(9):e0222547. doi: 10.1371/journal.pone.0222547 (PMC6743867; doi:10.1371/journal.pone.0222547)
Supplement: S2 Fig — (DOCX) [file pone.0222547.s002.docx]

S2 Fig. Correlation matrices between serum biomarkers of endothelial dysfunction and low-grade inflammation stratified by participant group

A. Controls


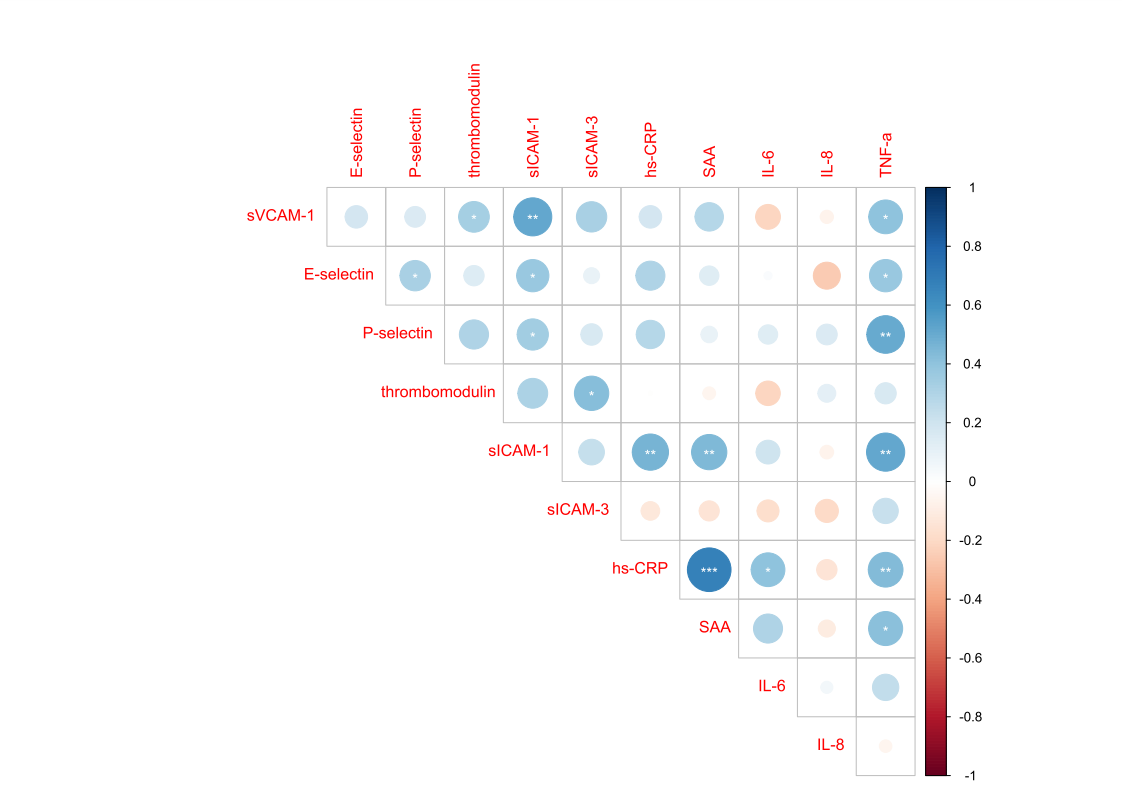


B. CKD5-ND


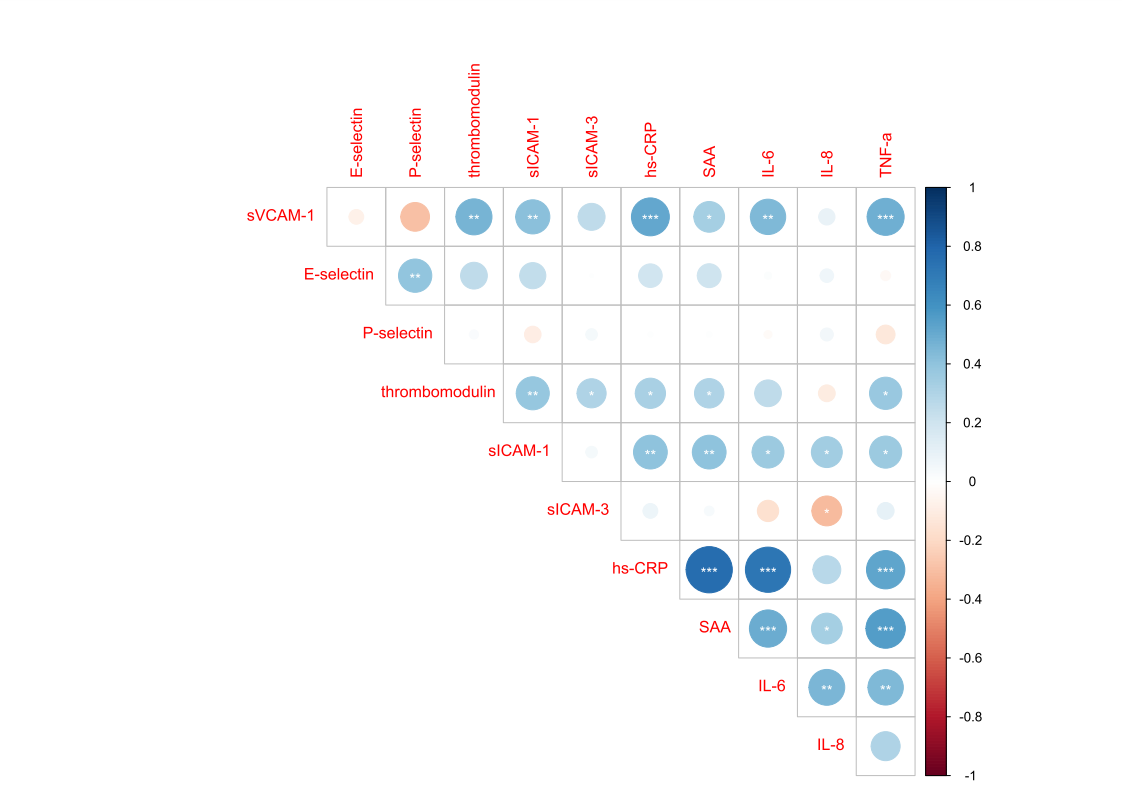


C. CKD5-HD


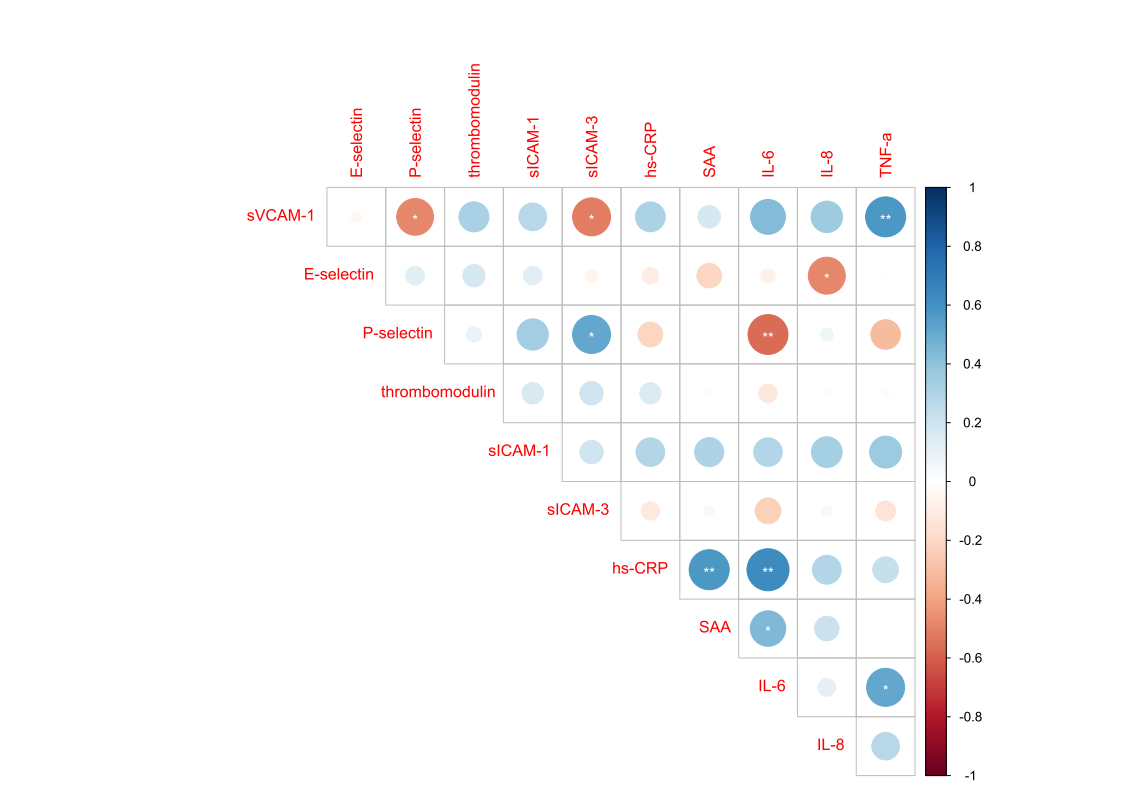


D: CKD5-PD


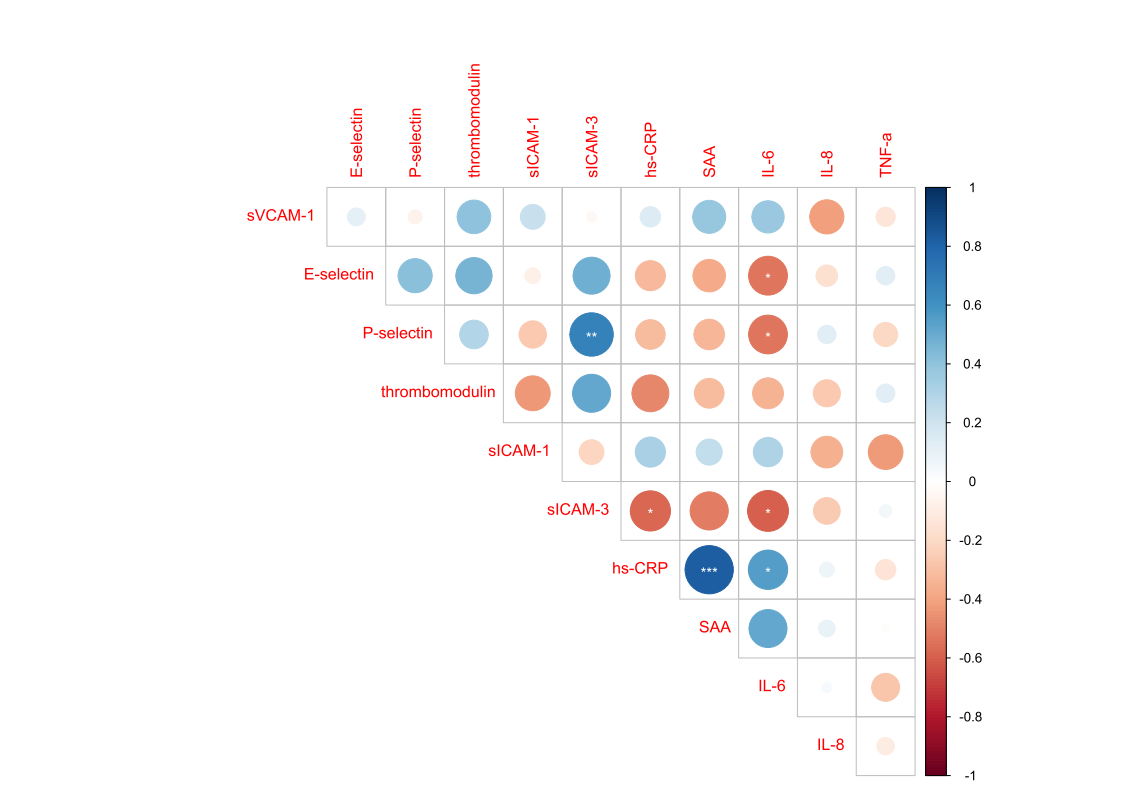


S2 Fig. Correlation matrices between serum biomarkers of endothelial dysfunction and low-grade inflammation stratified by participant group. Circle area and color indicate strength of Spearman’s rank correlation coefficients. Abbreviations:

CKD5-HD, chronic kidney disease stage 5 hemodialysis; CKD5-ND, chronic kidney disease stage 5 non-dialysis; CKD5-PD, chronic kidney disease stage 5 peritoneal dialysis; hs-CRP, high-sensitivity C-reactive protein; IL-6, interleukin 6; IL-8, interleukin 8; SAA, serum amyloid A; sICAM-1, soluble intercellular adhesion molecule 1; sICAM-3, soluble intercellular adhesion molecule 3; sVCAM-1, soluble vascular cell adhesion molecule 1; TNF-α, tumor necrosis factor alpha. * *P* < 0.050, ** *P* < 0.010, *** *P* < 0.001.
