# Supplementary material for: Endothelial dysfunction and low-grade inflammation in the transition to renal replacement therapy
Source: PLoS One. 2019 Sep 13;14(9):e0222547. doi: 10.1371/journal.pone.0222547 (PMC6743867; doi:10.1371/journal.pone.0222547)
Supplement: S1 Methods — (DOCX) [file pone.0222547.s003.docx]

**S1 Methods**

***In- and exclusion criteria of the individual studies***

Incident dialysis patients were individuals with ESRD who would start with dialysis within one month. Measurements were performed maximum four weeks prior to the first dialysis session. Exclusion criteria for incident dialysis patients were: acute start of dialysis treatment, active symptomatic coronary artery disease or cardiac failure New York Heart Association (NYHA) class III or IV, active malignancies, active infections, and inability to provide informed consent. Incident dialysis patients were recruited from the following dialysis centers in the South East of the Netherlands: Maastricht University Medical Center+ Maastricht, Catharina Hospital Eindhoven, Viecuri Hospital Venlo, Zuyderland Medical Centre Sittard, and St. Laurentius Hospital Roermond (Ethical Committee study number NL33129.068.10). This study was conducted between February 2012 and July 2017.

Kidney transplant recipients were individuals with ESRD receiving a living donor kidney transplant, age ≥18 years, and ability to provide informed consent. These individuals were recruited from the pre-transplantation clinic at the Maastricht University Medical Center+ in the Netherlands (Ethical Committee study number NL43381.068.13). Baseline measurements were performed within five days before kidney transplantation, with the exception of one patient who was examined five weeks before kidney transplantation. This study was conducted between October 2013 and January 2018.

Prevalent dialysis patients participated in the Uremic Toxins, Cardiovascular Effects and Physical Activity in Intensive Hemodialysis (INTHEMO) study, an international multicenter study which has been performed in the southern part of the Netherlands (Maastricht University Medical Center+ and Zuyderland Heerlen Dialysis Unit), Belgium (Jessa Hospital Hasselt Dialysis Unit), and the United Kingdom (Manchester University NHS Foundation Trust). For the present study, only participants from the Netherlands (Ethical Committee study number NL35039.068.10) were included to improve comparability with the incident dialysis patients, kidney transplant recipients and healthy controls. Prevalent dialysis patients were ≥18 years and treated with hemodialysis (HD) or peritoneal dialysis (PD) treatment for at least six months. Exclusion criteria for the prevalent dialysis patients were similar to those of incident dialysis patients, with the addition of chronic antibiotic use and colectomy. This study was conducted between June 2012 and December 2017.

Healthy donors were individuals who were suitable for living renal donation, for example, no uncontrolled or severe hypertension, and/or diabetes mellitus, and able to provide informed consent. These individuals were recruited from the pre-transplantation clinic at the Maastricht University Medical Center+ in the Netherlands. Baseline measurements were performed within one month before donation. This study was conducted between October 2013 and January 2018.

Healthy controls were non-diabetic, non-smokers, not hypertensive (systolic blood pressure <170 mmHg and/or diastolic blood pressure <100 mmHg), and able to provide informed consent, and were recruited via advertisements at the university hospital. This study was conducted between February 2012 and July 2017.

***Dialysis therapy modalities***

Detailed data on renal replacement therapy modalities were collected retrospectively and could be retrieved for 12 incident HD patients, 12 incident PD patients, 15 prevalent HD patients and 13 prevalent PD patients.

In both incident and prevalent HD patients, dialysis was performed three times per week for about four hours, using ultrapure dialysates and mostly high-flux dialyzers with synthetic membranes (polysulfone membranes (from Fresenius Medical Care GmbH, Bad Homburg, Germany) in 5 incident HD patients and 9 prevalent HD patients; polyethersulfone membrane (from Nipro, Osaka, Japan) in 4 incident HD patients and 3 prevalent HD patients; polyarylethersulfone/ polyvinylpyrrolidone/ polyamide blend membranes (Gambro, Lund, Sweden) in 1 incident HD patient). In addition, 2 incident HD patients and 3 prevalent HD patients were on low-flux dialyzers with synthetic membranes (polyarylethersulfone/ polyvinylpyrrolidone/ polyamide blend membranes (Gambro) and polysulfone membranes (Fresenius Medical Care), respectively). No participants were treated with hemodiafiltration. Vascular access was via an arteriovenous shunt in 14 incident HD patients and all prevalent HD patients, whereas 2 incident HD patients had a central venous catheter.

In both incident and prevalent PD patients, only PD fluids with low glucose-derived degradation product (GDP) content were used (Physioneal^®^, Extraneal^®^, and Nutrineal^®^ from Baxter, Castlebar, Ireland). The glucose concentration was prescribed at the discretion of the treating physician.

***Immunosuppressive protocol in incident kidney transplant recipients***

The main features of our centre’s immunosuppressive protocol were as follows: all kidney transplant recipients started with tacrolimus (TAC) and mycophenolate mofetil (MMF). TAC was pre-operatively dosed as 0.1 mg/kg, and post-operatively dosed as twice daily 0.1 mg/kg. The target range was 15-20 ng/ml in weeks 1 and 2, 10-15 ng/ml in weeks 3 and 4, and 5-7 ng/ml thereafter. MMF was given 1000 mg twice daily, starting with 1000 mg preoperatively.

Additionally, all kidney transplant recipients received corticosteroids for 10 days: methylprednisolone 125 mg daily on days 0 and 1, and prednisolone 10 mg daily from day 2 up to day 10. Prednisolone was continued only in recipients with a high risk for rejection or in patients with an IgA as primary disease. In recipients with an acute rejection, prednisolone was continued or re-introduced at a dose of 10 mg/ day. After 1 month, the dose of prednisolone was tapered to 5 mg daily.

After three months MMF was stopped in patients with low or intermediate immunological risk and without rejection in the first 3 months. Six months post-transplantation 5 patient were on TAC-monotherapy, 3 patients on TAC based dual-therapy (TAC and MMF) and 7 patients on TAC-based triple therapy (TAC, MMF and prednisolone).

Three patients included in this study were simultaneously enrolled in a different study (TRANSFORM) (NCT01950819). Two of these three patients were randomized to receive tacrolimus, everolimus and prednisolone as triple therapy from time of kidney transplant. One of these two patients withdrew informed consent for further follow-up in our study in the first week after kidney transplantation for personal reasons. The other received everolimus throughout the entire study period. The third patient was randomized to receive tacrolimus (prograft), MMF and prednisolone as triple therapy from time of kidney transplant. This patient switched to the local immunosuppressive protocol of our centre after the 6 month follow up visit.

***Immunosuppressive regimen in participants with prior kidney transplantation***

For the 14 participants who were included in the cross-sectional analyses and had a history of kidney transplantation, data on their immunosuppressive regimen were retrospectively collected from the electronic patient health record. Of the 8 participants with CKD5-ND and a history of kidney transplantation, 7 participants were on immunosuppressive therapy of whom the therapeutic regimen could be retrieved for 6 participants: 1 prednisolone monotherapy, 4 TAC monotherapy, 1 either TAC or MMF monotherapy (not entirely clear from the health record). In addition, in 1 participant, use of immunosuppressive medication could not be retrieved and in 1 participant with known use of immunosuppressive medication, the exact regimen could not be retrieved. Of the 3 participants with CKD5-HD and a history of kidney transplantation, 1 was on TAC monotherapy, 1 on MMF monotherapy and 1 did not use immunosuppressive therapy. Similarly, of the 3 participants with CKD5-PD and a history of kidney transplantation, 1 was one TAC monotherapy, 1 on MMF monotherapy and 1 did not use immunosuppressive therapy.

All incident HD patients with a history of kidney transplantation were on immunosuppressive medication: 2 TAC monotherapy from baseline throughout follow-up, 1 either TAC or MMF monotherapy (not entirely clear from the health record) from baseline up to and including the six month follow-up measurement, and for 1 of these participants the exact immunosuppressive regimen could not be retrieved.

Similarly, all incident PD patients with a history of kidney transplantation were on immunosuppressive medication: 1 prednisolone from baseline throughout follow-up and 1 TAC monotherapy from baseline up to and including the six months follow-up measurement.

All kidney transplant recipients with a history of kidney transplantation were on immunosuppressive therapy: 1 TAC monotherapy and 2 MMF monotherapy.
