# Supplementary material for: Endothelial dysfunction and low-grade inflammation in the transition to renal replacement therapy
Source: PLoS One. 2019 Sep 13;14(9):e0222547. doi: 10.1371/journal.pone.0222547 (PMC6743867; doi:10.1371/journal.pone.0222547)
Supplement: S1 Table — (DOCX) [file pone.0222547.s004.docx]

S1 Table. Serum biomarkers of endothelial dysfunction and low-grade inflammation at baseline

|  | Controls | CKD5-ND | CKD5-HD | CKD5-PD |
| --- | --- | --- | --- | --- |
|  | (n = 36) | (n = 43) | (n = 20) | (n = 14) |
| sVCAM-1 (μg/L) | 511.0 [452.3-555.0] | 887.0 [690.0-1,063.0] | 893.5 [725.3-997.5] | 1,064.0 [951.3-1,265.3] |
| E-selectin (μg/L) | 9.0 [6.5-10.9] | 11.9 [7.8-16.3] | 11.9 [7.2-15.1] | 17.0 [14.7-20.1] |
| P-selectin (μg/L) | 44.8 [35.3-59.0] | 51.6 [37.2-63.0] | 50.4 [37.6-64.4] | 47.4 [35.9-53.3] |
| Thrombomodulin (μg/L) | 2.9 [2.3-3.3] | 11.7 [9.5-13.8] | 12.0 [10.0-13.9] | 15.9 [12.1-20.0] |
| sICAM-1 (μg/L) | 363.5 [302.3-404.0] | 415.0 [373.0-504.0] | 392.5 [337.3-465.8] | 460.5 [380.3-508.0] |
| sICAM-3 (μg/L) | 0.9 [0.7-1.1] | 1.0 [0.8-1.3] | 0.9 [0.7-1.4] | 1.4 [1.0-1.7] |
| hs-CRP (mg/L) | 1.1 [0.5-2.3] | 3.2 [1.2-7.6] | 2.8 [1.7-15.5] | 4.0 [1.0-15.4] |
| SAA (mg/L) | 2.7 [1.4-5.7] | 6.8 [2.7-14.0] | 9.4 [4.0-25.9] | 12.5 [2.9-35.2] |
| IL-6 (ng/L) | 0.7 [0.5-0.9] | 1.6 [0.7-2.6] | 1.5 [1.1-2.4] | 1.6 [1.3-2.7] |
| IL-8 (ng/L) | 11.8 [9.5-15.6] | 13.8 [11.4-18.4] | 14.5 [10.6-17.1] | 10.9 [7.3-15.0] |
| TNF-α (ng/L) | 2.2 [2.0-2.3] | 5.0 [4.2-6.0] | 5.5 [4.5-7.0] | 5.8 [5.0-6.6] |
| Endothelial dysfunction Z-score | -0.98 ±0.73 | 0.36 ±0.68 | 0.24 ±0.76 | 1.05 ±0.72 |
| Low-grade inflammation Z-score | -0.88 ±0.74 | 0.37 ±0.92^†^ | 0.36 ±0.80 | 0.61 ±0.58 |

Data are presented median [25^th^ percentile – 75^th^ percentile].

Abbreviations: CKD5-HD, chronic kidney disease stage 5 hemodialysis; CKD5-ND, chronic kidney disease stage 5 non-dialysis; CKD5-PD, chronic kidney disease stage 5 peritoneal dialysis; hs-CRP, high-sensitivity C-reactive protein; IL-6, interleukin 6; IL-8, interleukin 8; SAA, serum amyloid A; sICAM-1, soluble intercellular adhesion molecule 1; sICAM-3, soluble intercellular adhesion molecule 3; sVCAM-1, soluble vascular cell adhesion molecule 1; TNF-α, tumor necrosis factor alpha.
