# Supplementary material for: Endothelial dysfunction and low-grade inflammation in the transition to renal replacement therapy
Source: PLoS One. 2019 Sep 13;14(9):e0222547. doi: 10.1371/journal.pone.0222547 (PMC6743867; doi:10.1371/journal.pone.0222547)
Supplement: S2 Table — (DOCX) [file pone.0222547.s005.docx]

S2 Table. Population characteristics longitudinal analyses

|  | Incident hemodialysis | Incident peritoneal dialysis | Kidney transplant recipients |
| --- | --- | --- | --- |
|  | (n = 18) | (n = 16) | (n = 15) |
| *Clinical characteristics* | | | |
| Age (years) | 65.1 ±12.0 | 57.1 ±12.1 | 51.6 ±12.8 |
| Men | 15 (83.3%) | 9 (56.2%) | 9 (60.0%) |
| Origin of end-stage renal disease: |  |  |  |
| Nephrosclerosis | 3 (16.7%) | 3 (18.8%) | 0 (0.0%) |
| Glomerulosclerosis | 0 (0.0%) | 0 (0.0%) | 1 (6.7%) |
| Hypertensive nephropathy | 1 (5.6%) | 1 (6.3%) | 1 (6.7%) |
| Renovascular disease | 0 (0.0%) | 0 (0.0%) | 0 (0.0%) |
| Diabetic nephropathy | 1 (5.6%) | 1 (6.3%) | 1 (6.7%) |
| Polycystic kidney disease | 5 (27.8%) | 4 (25.0%) | 4 (26.7%) |
| IgA nephropathy | 1 (5.6%) | 0 (0.0%) | 3 (20.0%) |
| Glomerulonephritis | 2 (11.1%) | 1 (6.3%) | 2 (13.3%) |
| Nephrotic syndrome | 3 (16.7%) | 2 (12.5%) | 1 (6.7%) |
| Other | 0 (0.0%) | 1 (6.3%) | 0 (0.0%) |
| Unknown | 2 (11.1%) | 3 (18.8%) | 2 (13.3%) |
| KTx treatment modality |  |  |  |
| Preemptive KTx | NA | NA | 6 (40.0%) |
| Non-preemptive KTx | NA | NA | 9 (60.0%) |
| Prior dialysis modality |  |  |  |
| None | NA | NA | 6 (40.0%) |
| HD | NA | Na | 5 (33.3%) |
| PD | NA | NA | 4 (26.7%) |
| History of KTx | 4 (22.2%) | 2 (12.5%) | 3 (20.0%) |
| Immunosuppressive therapy at baseline in participants with positive history of KTx* |  |  |  |
| Prednisolone monotherapy | 0 (0.0%) | 1 (50.0%) | 0 (0.0%) |
| TAC monotherapy | 2 (50.0%) | 1 (50.0%) | 1 (33.3%) |
| MMF monotherapy | 0 (0.0%) | 0 (0.0%) | 2 (66.7%) |
| TAC/MMF monotherapy (unclear) | 1 (25.0%) | 0 (0.0%) | 0 (0.0%) |
| Unknown | 1 (25.0%) | 0 (0.0%) | 0 (0.0%) |
| Serum creatinine (μmol/L)** | 616 [450-817] | 533 [421-622] | 542 [439-780] |
| eGFR_CKD-EPI_ (mL/min/1.73m^2^)** | 7.6 ±2.7 | 9.0 ±3.0 | 8.9 ±3.9 |
| Diuresis / Residual urine output** | 16 (100%) | 14 (100%) | 15 (100.0%) |
| Diuresis / Residual urine output (mL/24h)** | 2,100 [1,588-2,390] | 1,906 [1,800-2,215] | 1,500 [375-1,925] |
| Diabetes mellitus | 2 (11.1%) | 2 (12.5%) | 2 (13.3%) |
| Cardiovascular disease | 7 (38.9%) | 5 (31.2%) | 2 (13.3%) |
| Current smoking | 3 (16.7%) | 5 (31.2%) | 8 (23.5%) |
| BMI (kg/m^2^) | 26.6 ±3.8 | 23.5 ±3.0^¶^ | 24.5 ±4.7 |
| Fluid overload (L) | 1.9 ±2.4 | 0.8 ±1.4 | 0.4 ±2.2 |
| SBP (mmHg) | 146.2 ±22.3 | 150.1 ±28.0 | 148.9 ±27.2 |
| DBP (mmHg) | 77.4 ±9.2 | 87.3 ±16.0^¶^ | 86.7 ±12.5 |
| Renin-angiotensin-aldosterone system inhibitor use | 6 (33.3%) | 7 (43.8%) | 10 (66.7%) |
| Statin use | 11 (61.1%) | 8 (43.8%) | 5 (33.3%) |

Data are presented as n (%), mean ± standard deviation, or median [25^th^ percentile – 75^th^ percentile].

Abbreviations: BMI, body mass index; DBP, diastolic blood pressure; eGFR_CKD-EPI_, estimated glomerular filtration rate based on the creatinine CKD-EPI equation; eGFR_residual_, estimated residual GFR based on β2-microglobulin; HD, hemodialysis; KTx, kidney transplantation; MMF, mycophenolate mofetil; NA, not applicable; PD, peritoneal dialysis; SBP, systolic blood pressure; TAC, tacrolimus.

* More detailed information on immunosuppressive medication use is available in S1 Methods.

** Available in (Incident hemodialysis patients/ Incident peritoneal dialysis patients/ Kidney transplant recipients) n = 18/15/15 for serum creatinine n = 18/15/15 for eGFR_CKD-EPI_, n = 16/14/15 for residual urine output (dichotomous), n = 13/8/13 for residual urine output (continuous).

^¶^ *P* value < 0.05 vs. CKD5-HD based on Student’s t test for normally distributed data, Wilcoxon rank sum test for non-normally distributed data and Fisher’s exact test for categorical data.
