# Supplementary material for: Endothelial dysfunction and low-grade inflammation in the transition to renal replacement therapy
Source: PLoS One. 2019 Sep 13;14(9):e0222547. doi: 10.1371/journal.pone.0222547 (PMC6743867; doi:10.1371/journal.pone.0222547)
Supplement: S3 Table — (DOCX) [file pone.0222547.s006.docx]

S3 Table. Courses of serum biomarkers of endothelial dysfunction and low-grade inflammation stratified by dialysis modality

| Hemodialysis* | Time since dialysis initiation (months) | |
| --- | --- | --- |
| Serum biomarkers | 0 | 6 |
| sVCAM-1 | 856.5 [739.5-1,103.0] | 965.0 [740.8-1,138.0] |
| E-selectin | 12.1 [8.1-16.3] | 12.0 [7.2-15.0] |
| P-selectin | 53.3 [33.2-60.4] | 57.1 [50.3-77.6] |
| Thrombomodulin | 10.8 [9.7-13.5] | 11.4 [10.3-13.8] |
| sICAM-1 | 414.5 [382.8-511.8] | 432.0 [374.8-465.8] |
| sICAM-3 | 1.0 [0.8-1.3] | 1.0 [0.8-1.5] |
| hs-CRP | 4.8 [1.8-16.9] | 2.8 [1.7-6.1] |
| SAA | 8.5 [5.9-31.1] | 5.9 [3.3-9.3] |
| IL-6 | 1.8 [1.0-3.5] | 1.6 [1.0-2.0] |
| IL-8 | 14.0 [11.2-20.9] | 15.9 [10.8-20.9] |
| TNF-α | 4.9 [4.2-6.1] | 5.7 [5.2-6.7] |
|  |  |  |
| Peritoneal dialysis* | Time since dialysis initiation (months) | |
| Serum biomarkers | 0 | 6 |
| sVCAM-1 | 850.0 [666.0-1,110.8] | 912.5 [747.5-1,224.3] |
| E-selectin | 13.4 [10.1-17.5] | 15.3 [9.3-19.3] |
| P-selectin | 46.8 [42.0-62.5] | 48.4 [38.9-64.2] |
| Thrombomodulin | 12.2 [9.1-14.1] | 13.1 [10.7-17.7] |
| sICAM-1 | 468.5 [387.5-511.8] | 531.5 [417.8-584.3] |
| sICAM-3 | 0.9 [0.7-1.2] | 1.2 [0.9-1.3] |
| hs-CRP | 2.1 [0.9-5.5] | 2.3 [1.0-9.6] |
| SAA | 4.2 [2.6-8.6] | 4.2 [2.3-9.5] |
| IL-6 | 1.1 [0.7-1.9] | 1.4 [0.9-1.8] |
| IL-8 | 14.5 [12.0-18.2] | 12.9 [10.6-17.3] |
| TNF-α | 4.8 [4.1-5.6] | 5.4 [4.7-5.7] |

Data are presented as median [25^th^ percentile – 75^th^ percentile].

Abbreviations: hs-CRP, high-sensitivity C-reactive protein; IL-6, interleukin 6; IL-8, interleukin 8; NA, not applicable; SAA, serum amyloid A; sICAM-1, soluble intercellular adhesion molecule 1; sICAM-3, soluble intercellular adhesion molecule 3; sVCAM-1, soluble vascular cell adhesion molecule 1; TNF-α, tumor necrosis factor alpha.

* Analyses based on (hemodialysis/ peritoneal dialysis) n = 18/16.
