# Supplementary material for: Endothelial dysfunction and low-grade inflammation in the transition to renal replacement therapy
Source: PLoS One. 2019 Sep 13;14(9):e0222547. doi: 10.1371/journal.pone.0222547 (PMC6743867; doi:10.1371/journal.pone.0222547)
Supplement: S4 Table — (DOCX) [file pone.0222547.s007.docx]

S4 Table. Courses of serum biomarkers of endothelial dysfunction and low-grade inflammation following kidney transplantation

| Kidney transplant recipients* | Time since kidney transplantation (months) | | |
| --- | --- | --- | --- |
| Serum biomarkers | 0 | 3 | 6 |
| sVCAM-1 (μg/L) | 836.0 [763.0-929.0] | 644.0 [530.0-788.0] | 628.0 [574.0-715.0] |
| E-selectin (μg/L) | 11.1 [6.9-15.2] | 6.8 [5.0-17.7] | 9.3 [5.7-12.8] |
| P-selectin (μg/L) | 52.5 [41.8-71.6] | 51.3 [46.0-62.4] | 66.7 [48.3-82.5] |
| Thrombomodulin (μg/L) | 12.2 [9.6-13.9] | 4.6 [3.6-5.5] | 5.0 [4.1-5.4] |
| sICAM-1 (μg/L) | 372.0 [331.0-470.0] | 387.0 [277.0-462.0] | 368.0 [303.0-424.0] |
| sICAM-3 (μg/L) | 1.4 [1.1-1.5] | 1.0 [0.8-1.1] | 1.1 [0.8-1.2] |
| hs-CRP (mg/L) | 4.1 [1.6-20.5] | 1.6 [0.6-3.1] | 1.5 [0.7-7.9] |
| SAA (mg/L) | 9.4 [2.7-76.4] | 6.8 [2.9-16.5] | 5.4 [2.4-21.9] |
| IL-6 (ng/L) | 1.3 [0.7-2.1] | 0.9 [0.6-1.3] | 1.0 [0.6-1.6] |
| IL-8 (ng/L) | 10.9 [7.4-14.2] | 11.3 [7.3-18.2] | 12.6 [8.8-28.5] |
| TNF-α (ng/L) | 5.8 [4.4-6.9] | 3.0 [2.6-4.1] | 3.2 [2.6-4.1] |

Data are presented as median [25^th^ percentile – 75^th^ percentile].

Abbreviations: hs-CRP, high-sensitivity C-reactive protein; IL-6, interleukin 6; IL-8, interleukin 8; NA, not applicable; SAA, serum amyloid A; sICAM-1, soluble intercellular adhesion molecule 1; sICAM-3, soluble intercellular adhesion molecule 3; sVCAM-1, soluble vascular cell adhesion molecule 1; TNF-α, tumor necrosis factor alpha.

* Analyses based on n = 15.
