# Supplementary material for: Endothelial dysfunction and low-grade inflammation in the transition to renal replacement therapy
Source: PLoS One. 2019 Sep 13;14(9):e0222547. doi: 10.1371/journal.pone.0222547 (PMC6743867; doi:10.1371/journal.pone.0222547)
Supplement: S5 Table — (DOCX) [file pone.0222547.s008.docx]

S5 Table. Associations of end-stage renal disease with serum biomarkers of endothelial dysfunction and low-grade inflammation after exclusion of individuals with standardized residuals smaller than -2 or larger than 2 standard deviations

|  | CKD5-ND vs. controls* | | CKD5-HD vs. controls* | | CKD5-PD vs. controls* | |
| --- | --- | --- | --- | --- | --- | --- |
| **Individual serum biomarkers** | **Ratio (95%CI)** | ***P* value** | **Ratio (95%CI)** | ***P* value** | **Ratio (95%CI)** | ***P* value** |
| sVCAM-1 | 1.65 (1.50; 1.82) | < 0.001 | 1.65 (1.45; 1.87) | < 0.001 | 2.04 (1.78; 2.35) | < 0.001 |
| E-selectin | 1.32 (1.12; 1.57) | 0.001 | 1.21 (0.97; 1.52) | 0.092 | 1.59 (1.23; 2.06) | < 0.001 |
| P-selectin | 1.12 (0.95; 1.31) | 0.182 | 1.19 (0.96; 1.48) | 0.103 | 0.98 (0.78; 1.23) | 0.840 |
| Thrombomodulin | 4.15 (3.74; 4.60) | < 0.001 | 4.29 (3.75; 4.60) | < 0.001 | 5.60 (4.80; 6.55) | < 0.001 |
| sICAM-1 | 1.21 (1.10; 1.32) | < 0.001 | 1.11 (0.99; 1.25) | 0.076 | 1.25 (1.10; 1.43) | 0.001 |
| sICAM-3 | 1.12 (0.96; 1.32) | 0.146 | 1.06 (0.86; 1.31) | 0.590 | 1.53 (1.22; 1.93) | < 0.001 |
| hs-CRP | 2.95 (1.68; 5.17) | < 0.001 | 3.83 (1.84; 7.97) | < 0.001 | 3.80 (1.70; 8.50) | 0.001 |
| SAA | 2.19 (1.36; 3.54) | 0.002 | 4.19 (2.26; 7.75) | < 0.001 | 4.00 (1.94; 8.24) | < 0.001 |
| IL-6 | 1.99 (1.53; 2.59) | < 0.001 | 2.03 (1.45; 2.86) | < 0.001 | 2.29 (1.56; 3.36) | < 0.001 |
| IL-8 | 1.22 (1.00; 1.50) | 0.055 | 1.21 (0.92; 1.58) | 0.174 | 0.96 (0.72; 1.29) | 0.793 |
| TNF-α | 2.21 (2.01; 2.42) | < 0.001 | 2.59 (2.30; 2.92) | < 0.001 | 2.63 (2.29; 3.01) | < 0.001 |
| **Z-scores** | **Beta (95%CI)** | ***P* value** | **Beta (95%CI)** | ***P* value** | **Beta (95%CI)** | ***P* value** |
| Endothelial dysfunction | 1.45 (1.15; 1.76) | < 0.001 | 1.31 (0.92; 1.71) | < 0.001 | 2.11 (1.68; 2.54) | < 0.001 |
| Low-grade inflammation | 1.17 (0.86; 1.47) | < 0.001 | 1.25 (0.85; 1.65) | < 0.001 | 1.63 (1.20; 2.07) | < 0.001 |
|  |  |  |  |  |  |  |
|  | CKD5-HD vs. CKD5-ND* | | CKD5-PD vs. CKD5-ND* | | CKD5-PD vs. CKD5-HD* | |
| **Individual serum biomarkers** | **Ratio (95%CI)** | ***P* value** | **Ratio (95%CI)** | ***P* value** | **Ratio (95%CI)** | ***P* value** |
| sVCAM-1 | 1.00 (0.88; 1.12) | 0.953 | 1.24 (1.08; 1.41) | 0.002 | 1.24 (1.07; 1.44) | 0.005 |
| E-selectin | 0.92 (0.74; 1.13) | 0.423 | 1.20 (0.94; 1.54) | 0.136 | 1.31 (1.00; 1.72) | 0.049 |
| P-selectin | 1.07 (0.88; 1.30) | 0.498 | 0.88 (0.71; 1.09) | 0.225 | 0.82 (0.64; 1.04) | 0.102 |
| Thrombomodulin | 1.03 (0.91; 1.17) | 0.603 | 1.35 (1.17; 1.57) | < 0.001 | 1.31 (1.11; 1.53) | 0.001 |
| sICAM-1 | 0.92 (0.83; 1.03) | 0.157 | 1.04 (0.92; 1.18) | 0.556 | 1.12 (0.98; 1.29) | 0.100 |
| sICAM-3 | 0.94 (0.77; 1.15) | 0.565 | 1.36 (1.10; 1.69) | 0.005 | 1.45 (1.13; 1.85) | 0.003 |
| hs-CRP | 1.30 (0.65; 2.59) | 0.456 | 1.29 (0.60; 2.78) | 0.516 | 0.99 (0.43; 2.30) | 0.985 |
| SAA | 1.91 (1.06; 3.44) | 0.031 | 1.82 (0.91; 3.66) | 0.090 | 0.95 (0.45; 2.01) | 0.901 |
| IL-6 | 1.02 (0.74; 1.41) | 0.888 | 1.15 (0.80; 1.66) | 0.454 | 1.12 (0.76; 1.67) | 0.560 |
| IL-8 | 0.98 (0.76; 1.27) | 0.903 | 0.79 (0.59; 1.04) | 0.091 | 0.80 (0.58; 1.09) | 0.155 |
| TNF-α | 1.17 (1.05; 1.31) | 0.006 | 1.19 (1.05; 1.36) | 0.009 | 1.02 (0.88; 1.17) | 0.832 |
| **Z-scores** | **Beta (95%CI)** | ***P* value** | **Beta (95%CI)** | ***P* value** | **Beta (95%CI)** | ***P* value** |
| Endothelial dysfunction | -0.14 (-0.50; 0.22) | 0.454 | 0.65 (0.25; 1.06) | 0.002 | 0.79 (0.34; 1.24) | < 0.001 |
| Low-grade inflammation | 0.09 (-0.29; 0.46) | 0.650 | 0.47 (0.05; 0.88) | 0.029 | 0.38 (-0.08; 0.84) | 0.105 |

Ratios represent the ratio of (geometric mean) levels of the serum biomarkers in the respective end-stage renal disease group relative to controls, relative to individuals with chronic kidney disease stage 5 non-dialysis, chronic kidney disease stage 5 hemodialysis, and chronic kidney disease stage 5 peritoneal dialysis, respectively.

Betas represent the differences in Z-scores for endothelial dysfunction and low-grade inflammation (expressed as standard deviations) between the respective end-stage renal disease group and controls, and among the respective end-stage renal disease groups.

All analyses are adjusted for age, sex and diabetes mellitus.

Abbreviations: CKD5-HD, chronic kidney disease stage 5 hemodialysis; CKD5-ND, chronic kidney disease stage 5 non-dialysis; CKD5-PD, chronic kidney disease stage 5 peritoneal dialysis; hs-CRP, high-sensitivity C-reactive protein; IL-6, interleukin 6; IL-8, interleukin 8; SAA, serum amyloid A; sICAM-1, soluble intercellular adhesion molecule 1; sICAM-3, soluble intercellular adhesion molecule 3; sVCAM-1, soluble vascular cell adhesion molecule 1; TNF-α, tumor necrosis factor alpha.

* Outliers were defined as participants with standardized residuals < -2 or > 2 standard deviations in linear regression analyses on the respective serum biomarker.
