# Supplementary material for: Endothelial dysfunction and low-grade inflammation in the transition to renal replacement therapy
Source: PLoS One. 2019 Sep 13;14(9):e0222547. doi: 10.1371/journal.pone.0222547 (PMC6743867; doi:10.1371/journal.pone.0222547)
Supplement: S6 Table — (DOCX) [file pone.0222547.s009.docx]

S6 Table. Course of serum biomarkers of endothelial dysfunction and low-grade inflammation stratified by dialysis modality after exclusion of individuals with standardized residuals smaller than -2 or larger than 2 standard deviations

|  |  | Ratios of biomarker following dialysis initiation levels* | | |
| --- | --- | --- | --- | --- |
|  |  | 6 month vs. baseline | | |
| Biomarkers | Modality | Ratio (95%CI) | *P* value | *P*_interaction_** |
| sVCAM-1 (μg/L) | HD | 1.09 (1.02; 1.16) | 0.011 | 0.947 |
|  | PD | 1.09 (1.02; 1.17) | 0.011 |  |
| E-selectin (μg/L) | HD | 1.00 (0.88; 1.14) | 0.983 | 0.509 |
|  | PD | 1.07 (0.93; 1.23) | 0.355 |  |
| P-selectin (μg/L) | HD | 1.24 (1.10; 1.38) | < 0.001 | 0.015 |
|  | PD | 1.00 (0.89; 1.13) | 0.938 |  |
| Thrombomodulin (μg/L) | HD | 1.06 (0.98; 1.13) | 0.130 | 0.352 |
|  | PD | 1.11 (1.02; 1.20) | 0.013 |  |
| sICAM-1 (μg/L) | HD | 1.01 (0.93; 1.10) | 0.798 | 0.065 |
|  | PD | 1.13 (1.03; 1.23) | 0.007 |  |
| sICAM-3 (μg/L) | HD | 1.06 (0.98; 1.14) | 0.152 | 0.022 |
|  | PD | 1.20 (1.11; 1.30) | < 0.001 |  |
| hs-CRP (mg/L) | HD | 0.54 (0.36; 0.81) | 0.004 | 0.015 |
|  | PD | 1.14 (0.74; 1.74) | 0.543 |  |
| SAA (mg/L) | HD | 0.56 (0.38; 0.82) | 0.004 | 0.046 |
|  | PD | 0.99 (0.66; 1.51) | 0.973 |  |
| IL-6 (ng/L) | HD | 0.88 (0.70; 1.09) | 0.234 | 0.093 |
|  | PD | 1.15 (0.92; 1.44) | 0.225 |  |
| IL-8 (ng/L) | HD | 0.91 (0.74; 1.12) | 0.364 | 0.956 |
|  | PD | 0.90 (0.73; 1.11) | 0.325 |  |
| TNF-α (ng/L) | HD | 1.15 (1.07; 1.24) | < 0.001 | 0.668 |
|  | PD | 1.13 (1.04; 1.22) | 0.004 |  |

Ratios represent the ratio of (geometric mean) levels of the biomarkers at the respective time point after dialysis initiation relative to baseline levels based on a linear mixed model containing the respective serum biomarkers, categorical time, serum biomarker*categorical time, and a random intercept.

Abbreviations: hs-CRP, high-sensitivity C-reactive protein; IL-6, interleukin 6; IL-8, interleukin 8; NA, not applicable; SAA, serum amyloid A; sICAM-1, soluble intercellular adhesion molecule 1; sICAM-3, soluble intercellular adhesion molecule 3; sVCAM-1, soluble vascular cell adhesion molecule 1; TNF-α, tumor necrosis factor alpha.

* * Outliers were defined as participants with standardized residuals < -2 or > 2 standard deviations in linear mixed model analyses on the respective serum biomarker. Please note that there were not outliers for E-selectin, P-selectin, sICAM-3 and hs-CRP, and thus results were equal to the primary analyses.

** *P* value for the interaction term between categorical time and dialysis modality.
