# Supplementary material for: Endothelial dysfunction and low-grade inflammation in the transition to renal replacement therapy
Source: PLoS One. 2019 Sep 13;14(9):e0222547. doi: 10.1371/journal.pone.0222547 (PMC6743867; doi:10.1371/journal.pone.0222547)
Supplement: S7 Table — (DOCX) [file pone.0222547.s010.docx]

S7 Table. Courses of serum biomarkers of endothelial dysfunction and low-grade inflammation following kidney transplantation after exclusion of individuals with standardized residuals smaller than -2 or larger than 2 standard deviations

| Kidney transplant recipients | Ratios of biomarkers following kidney transplantation* | | | |
| --- | --- | --- | --- | --- |
|  | 3 months vs. baseline | | 6 months vs. baseline | |
| Serum biomarkers | Ratio (95%CI) | *P* value | Ratio (95%CI) | *P* value |
| sVCAM-1 (μg/L) | 0.72 (0.64; 0.82) | < 0.001 | 0.76 (0.67; 0.86) | < 0.001 |
| E-selectin (μg/L) | 0.76 (0.63; 0.92) | 0.008 | 0.82 (0.79; 1.00) | 0.049 |
| P-selectin (μg/L) | 1.08 (0.95; 1.21) | 0.227 | 1.22 (1.08; 1.38) | 0.002 |
| Thrombomodulin (μg/L) | 0.42 (0.37; 0.47) | < 0.001 | 0.43 (0.38; 0.48) | < 0.001 |
| sICAM-1 (μg/L) | 0.89 (0.78; 1.01) | 0.065 | 0.91 (0.80; 1.04) | 0.149 |
| sICAM-3 (μg/L) | 0.69 (0.60; 0.79) | < 0.001 | 0.77 (0.67; 0.88) | < 0.001 |
| hs-CRP (mg/L) | 0.36 (0.15; 0.86) | 0.022 | 0.56 (0.24; 1.35) | 0.193 |
| SAA (mg/L) | 0.56 (0.24; 1.32) | 0.175 | 0.66 (0.28; 1.54) | 0.321 |
| IL-6 (ng/L) | 0.83 (0.55; 1.24) | 0.346 | 0.80 (0.54; 1.21) | 0.279 |
| IL-8 (ng/L) | 1.14 (0.74; 1.74) | 0.539 | 1.70 (1.11; 2.60) | 0.016 |
| TNF-α (ng/L) | 0.63 (0.52; 0.76) | < 0.001 | 0.60 (0.50; 0.72) | < 0.001 |

Ratios represent the ratio of (geometric mean) levels of the biomarkers at the respective time point after kidney transplantation relative to baseline levels based on a linear mixed model containing categorical time and a random intercept.

Abbreviations: hs-CRP, high-sensitivity C-reactive protein; IL-6, interleukin 6; IL-8, interleukin 8; NA, not applicable; SAA, serum amyloid A; sICAM-1, soluble intercellular adhesion molecule 1; sICAM-3, soluble intercellular adhesion molecule 3; sVCAM-1, soluble vascular cell adhesion molecule 1; TNF-α, tumor necrosis factor alpha.

* Outliers were defined as participants with standardized residuals < -2 or > 2 standard deviations in linear mixed model analyses on the respective serum biomarker.
