# Supplementary material for: Endothelial dysfunction and low-grade inflammation in the transition to renal replacement therapy
Source: PLoS One. 2019 Sep 13;14(9):e0222547. doi: 10.1371/journal.pone.0222547 (PMC6743867; doi:10.1371/journal.pone.0222547)
Supplement: S8 Table — (DOCX) [file pone.0222547.s011.docx]

S8 Table. Courses of serum biomarkers of endothelial dysfunction and low-grade inflammation following kidney transplantation after exclusion of participants with a history of kidney transplantation

| Kidney transplant recipients | Ratios of biomarkers following kidney transplantation* | | | |
| --- | --- | --- | --- | --- |
|  | 3 months vs. baseline | | 6 months vs. baseline | |
| Serum biomarkers | Ratio (95%CI) | *P* value | Ratio (95%CI) | *P* value |
| sVCAM-1 (μg/L) | 0.81 (0.70; 0.94) | 0.009 | 0.75 (0.64; 0.87) | < 0.001 |
| E-selectin (μg/L) | 0.86 (0.66; 1.12) | 0.258 | 0.86 (0.66; 1.13) | 0.264 |
| P-selectin (μg/L) | 0.98 (0.77; 1.26) | 0.880 | 1.19 (0.93; 1.52) | 0.160 |
| Thrombomodulin (μg/L) | 0.39 (0.33; 0.46) | < 0.001 | 0.40 (0.34; 0.47) | < 0.001 |
| sICAM-1 (μg/L) | 0.98 (0.92; 1.18) | 0.839 | 0.89 (0.74; 1.07) | 0.218 |
| sICAM-3 (μg/L) | 0.74 (0.62; 0.87) | 0.001 | 0.81 (0.68; 0.96) | 0.018 |
| hs-CRP (mg/L) | 0.42 (0.15; 1.18) | 0.095 | 0.34 (0.12; 0.95) | 0.041 |
| SAA (mg/L) | 0.65 (0.21; 1.97) | 0.429 | 0.40 (0.13; 1.21) | 0.099 |
| IL-6 (ng/L) | 0.50 (0.33; 1.10) | 0.094 | 0.55 (0.30; 0.99) | 0.046 |
| IL-8 (ng/L) | 0.85 (0.43; 1.71) | 0.641 | 1.09 (0.54; 2.19) | 0.797 |
| TNF-α (ng/L) | 0.53 (0.41; 0.68) | < 0.001 | 0.48 (0.37; 0.63) | < 0.001 |

Ratios represent the ratio of (geometric mean) levels of the biomarkers at the respective time point after kidney transplantation relative to baseline levels based on a linear mixed model containing categorical time and a random intercept.

Abbreviations: hs-CRP, high-sensitivity C-reactive protein; IL-6, interleukin 6; IL-8, interleukin 8; NA, not applicable; SAA, serum amyloid A; sICAM-1, soluble intercellular adhesion molecule 1; sICAM-3, soluble intercellular adhesion molecule 3; sVCAM-1, soluble vascular cell adhesion molecule 1; TNF-α, tumor necrosis factor alpha.

* Analyses based on n = 12.
