# Supplementary material for: Endothelial dysfunction and low-grade inflammation in the transition to renal replacement therapy
Source: PLoS One. 2019 Sep 13;14(9):e0222547. doi: 10.1371/journal.pone.0222547 (PMC6743867; doi:10.1371/journal.pone.0222547)
Supplement: S9 Table — (DOCX) [file pone.0222547.s012.docx]

S9 Table. Course of serum biomarkers of endothelial dysfunction and low-grade inflammation stratified by dialysis modality after additional adjustment for estimated residual glomerular filtration rate

|  |  | Ratios of biomarker following dialysis initiation levels* | | |
| --- | --- | --- | --- | --- |
|  |  | 6 month vs. baseline | | |
| Biomarkers | Modality | Ratio (95%CI) | *P* value | *P*_interaction_** |
| sVCAM-1 (μg/L) | HD | 1.00 (0.92; 1.09) | 0.955 | 0.232 |
|  | PD | 1.06 (0.99; 1.16) | 0.089 |  |
| E-selectin (μg/L) | HD | 0.93 (0.79; 1.09) | 0.338 | 0.138 |
|  | PD | 1.08 (0.93; 1.24) | 0.299 |  |
| P-selectin (μg/L) | HD | 1.20 (1.05; 1.37) | 0.010 | 0.013 |
|  | PD | 0.96 (0.85; 1.09) | 0.519 |  |
| Thrombomodulin (μg/L) | HD | 1.09 (0.98; 1.20) | 0.102 | 0.077 |
|  | PD | 0.88 (0.79; 0.97) | 0.016 |  |
| sICAM-1 (μg/L) | HD | 0.93 (0.84; 1.02) | 0.136 | 0.010 |
|  | PD | 1.10 (1.01; 1.21) | 0.036 |  |
| sICAM-3 (μg/L) | HD | 1.04 (0.94; 1.15) | 0.449 | 0.025 |
|  | PD | 1.20 (1.10; 1.31) | < 0.001 |  |
| hs-CRP (mg/L) | HD | 0.42 (0.27; 0.65) | < 0.001 | 0.001 |
|  | PD | 1.12 (0.75; 1.68) | 0.560 |  |
| SAA (mg/L) | HD | 0.36 (0.21; 0.61) | < 0.001 | 0.008 |
|  | PD | 0.92 (0.57; 1.51) | 0.746 |  |
| IL-6 (ng/L) | HD | 0.69 (0.54; 0.88) | 0.004 | 0.003 |
|  | PD | 1.14 (0.91; 1.44) | 0.245 |  |
| IL-8 (ng/L) | HD | 0.98 (0.77; 1.26) | 0.895 | 0.550 |
|  | PD | 0.89 (0.70; 1.14) | 0.343 |  |
| TNF-α (ng/L) | HD | 1.25 (1.04; 1.50) | 0.021 | 0.403 |
|  | PD | 1.12 (0.93; 1.35) | 0.213 |  |

Ratios represent the ratio of (geometric mean) levels of the biomarkers at the respective time point after dialysis initiation relative to baseline levels based on a linear mixed model containing the respective serum biomarkers, categorical time, serum biomarker*categorical time, age, sex, diabetes mellitus, estimated residual glomerular filtration rate, and a random intercept.

Abbreviations: hs-CRP, high-sensitivity C-reactive protein; IL-6, interleukin 6; IL-8, interleukin 8; NA, not applicable; SAA, serum amyloid A; sICAM-1, soluble intercellular adhesion molecule 1; sICAM-3, soluble intercellular adhesion molecule 3; sVCAM-1, soluble vascular cell adhesion molecule 1; TNF-α, tumor necrosis factor alpha.

* Analyses based on (incident hemodialysis/ incident peritoneal dialysis) n = 18/16.

** *P* value for the interaction term between categorical time and dialysis modality.
